# Effect of Nut Consumption on Nonalcoholic Fatty Liver Disease: A Systematic Review and Meta-Analysis

**DOI:** 10.3390/nu15102394

**Published:** 2023-05-19

**Authors:** Ling Pan, Jing Sui, Ying Xu, Qun Zhao

**Affiliations:** 1 Research Institute for Environment and Health, Nanjing University of Information Science and Technology, Nanjing 210044, China; 2 Key Laboratory of Environmental Medicine Engineering, Ministry of Education, School of Public Health, Southeast University, Nanjing 210009, China

**Keywords:** nonalcoholic fatty liver disease, NAFLD, nut consumption, diet, meta-analysis

## Abstract

Although previous epidemiological studies have been conducted to investigate the relationship between nut consumption and the risk of nonalcoholic fatty liver disease (NAFLD), the evidence remains inconclusive and contentious. The aim of our study was to further conduct a meta-analysis of observational studies to explore the latest evidence of the influence of nut consumption on NAFLD. This meta-analysis included a comprehensive search of all articles published in the PubMed and Web of Science online databases as of April 2023. A total of 11 articles were included, comprising 2 prospective cohort studies, 3 cross-sectional studies, and 7 case–control studies, and a random effects model was used to evaluate the relationship between nuts and NAFLD. Results showed that the odds ratio (OR) of NAFLD was 0.90 (95% CI: 0.81–0.99, *p* < 0.001) when comparing the highest and lowest total nut intake, indicating a significant negative correlation. Furthermore, subgroup analysis revealed that the protective effect of nuts on NAFLD was more significant in females (OR = 0.88; 95% CI: 0.78–0.98, *I*^2^ = 76.2%). In summary, our findings provide support for a protective relationship between nut intake and risk of NAFLD. Further exploration of the association between other dietary components and NAFLD is an important avenue for future research.

## 1. Introduction

Nonalcoholic fatty liver disease (NAFLD) is a significant public health concern and is the primary cause of chronic liver disease. NAFLD is a liver manifestation of metabolic syndrome and is prevalent in obese and diabetic patients [1]. With the rising incidence of obesity and diabetes worldwide, NAFLD is becoming increasingly prevalent. Currently, pharmacological treatments for NAFLD lack both safety and efficacy, and lifestyle interventions such as weight loss, a balanced diet, and physical exercise remain among the best treatments for NAFLD [2]. A growing number of studies have shown a negative correlation between the Mediterranean diet and the incidence of NAFLD [3]. The Mediterranean diet, characterized by a high content of polyphenols, carotenoids, vitamins, and polyunsaturated fats, has anti-inflammatory and antioxidant effects, and has been shown to be highly effective in reducing the risk of metabolic syndrome [4].

Metabolic-associated fatty liver disease (MAFLD) is a novel concept proposed in 2020. Nut consumption is a crucial part of the Mediterranean diet and an important contributor of plant white matter, unsaturated fat acid (UFA), dietary fibers (DF), antioxidants and polyphenols, protein, vitamin E, and some trace elements (such as zinc and selenium) [5,6]. Nuts are widely available worldwide and comprise hard shells and edible cores. Tree nuts, such as almonds, walnuts, and pistachios, possess rich nutritional functions and are beneficial to various health outcomes [7]. A review showed that DF can reduce liver exposure to bacterial products such as lipopolysaccharide and positively affect liver health [8]. An animal study indicated that increasing dietary fiber intake can effectively reduce serum pro-inflammatory factors, such as tumor necrosis factor alpha (TNF-α), interleukin (IL) 1-β, and effectively alleviate liver fibrosis [9]. In addition to dietary fiber, protein and UFA have beneficial effects on alleviating liver fibrosis [10,11]. In a randomized controlled study, a significant decrease in liver lipid content was observed in a group receiving large amounts of single/multiple UFA, which had a positive impact on liver fat and lipid metabolism [12]. Vitamin E has lipid peroxidation inhibitory activity and can prevent NAFLD [13]. The liver is the main organ responsible for zinc metabolism, and a lack of zinc may alter liver cell function and immune response in inflammatory liver disease, leading to cirrhosis [14]. Tang et al. [15] found that the lack of trace element selenium may cause redox imbalance and lead to liver inflammation.

Nut consumption has a protective effect on cognitive function, particularly among individuals at high risk of cognitive decline [16]. Naghshi et al. demonstrated that nut consumption was related to the reduction in mortality and morbidity of different cancers [17]. A case–control study involving 39,167 healthy women found a negative correlation between nut consumption and cardiovascular mortality in females. Compared to nonconsumers, the risk ratio of cardiovascular mortality was 0.73 (0.61–0.87) for nut intake ≥ 2 times a week (*p* = 0.0004) [18].

Another cross-sectional study revealed that moderate intake of nuts and seeds significantly reduced the incidence of NAFLD in males (9%) and females (15%) compared with nonconsumers [19]. Recent epidemiological studies have found a beneficial effect of nut consumption on NAFLD [19,20]. Zhang et al. [21] found that the higher nut intake, the less incidence of NAFLD (OR = 0.80; 95% CI: 0.69–0.92). In a case–control study (134 cases and 217 controls), the results showed that nuts had a beneficial influence on NAFLD (OR = 0.95; 95% CI: 0.91–0.98) [22]. However, three studies have reported a reverse relationship between nuts and NAFLD [23,24,25]. In a study targeting individuals aged ≥25 in India, it was reported that consuming nuts poses a risk of NAFLD (OR = 1.21; 95% CI: 1.12–1.52) [25].

Previous meta-analysis has explored the relationship between nut consumption and the risk of various diseases, such as diabetes, obesity, and cancer [26,27,28]. However, no meta-analysis to date has specifically summarized the relationship between nut consumption and the risk of NAFLD. Therefore, further analysis is warranted to consolidate these research findings. In this study, we used individual nut intake as an exposure variable to clarify the relationship between nut consumption and risk of NAFLD. Our meta-analysis aims to provide novel evidence for a comprehensive assessment of the link between nut consumption and NAFLD risk by utilizing existing studies.

## 2. Materials and Methods

The design and reporting in our study followed the Preferred Reporting Items for Systematic Reviews and Meta-Analyses (PRISMA) [29]. This work was registered with PROSPERO (CDR42023423356).

### 2.1. Search Strategy

To identify observational studies on the relationship between nut intake and NAFLD, we conducted a systematic search of the PubMed and Web of Science databases for articles published online prior to April 2023. Metabolic-dysfunction-associated fatty liver disease (MAFLD) is a novel concept proposed in 2020 [30]. We also included the concept in search terms. The search terms used included “Nuts”, “Almonds”, “Cashew Nuts”, “Peanuts”, “Pecans”, “Pinenuts”, “Pistachios”, “Nut Tree”, “Macadamia Nuts”, “Hazelnuts”, “Walnuts”, “Fatty Liver”, “Steatohepatitis”, “Steatosis of Liver”, “Steatohepatitides”, “Nonalcoholic Fatty Liver Disease”, “NAFLD”,” Metabolic Dysfunction-Associated Fatty Liver Disease”, “MAFLD”. This study restricted the search to publications in English. We also searched the reference list of the original article to avoid missing relevant research.

### 2.2. Inclusion and Exclusion Criteria

The criteria for inclusion in this study were as follows: (1) observational epidemiological studies, cohort studies, case–control studies, or cross-sectional studies; (2) the correlation between nut intake and the risk of NAFLD; (3) data such as hazard ratio (HR), risk ratio (RR), or odds ratio (OR), and a reported confidence interval (CI) of 95%; (4) the intake of nuts (peanuts, walnuts, pistachios, macadamia nuts, hickories, cashews, almonds, hazelnuts, and Brazil nuts) as exposure variables and the risk of NAFLD as the outcome variable. If more than 1 dataset was published in different articles, we selected the latest version for analysis. When referring to articles, we excluded the following: (1) letters, reviews, case reports, and comments; (2) studies that did not contain nut intake; (3) studies related to animals and cells; (4) studies that did not contain HR, RR, or OR [17,31].

### 2.3. Data Extraction

L.P. and Y.X., independently screened the title, abstract, and full text of the study, retaining articles that met the criteria. Any differences were resolved through discussion, mediation, and consultation with a third researcher (J.S.). From each study, the year of publication, the name of the first author, the type of study, the duration of the study, the location of the study, the number of participants, their gender, confounding variables, and the OR, HR, or RR between nut intake and NAFLD risk, as well as the corresponding 95% CI, were extracted.

### 2.4. Quality Assessment

Quality was assessed using the Newcastle-Ottawa Scale (NOS) scored on a scale of 0 to 9 [32]. The scoring parameters are as follows: participants choose up to 4 points, comparability may have up to 2 points, and result evaluation may have up to 3 points [17]. The study with a score above the average is considered to be of high quality.

### 2.5. Statistical Analysis 

This study used OR or RR or HR to represent the magnitude of the effect. The random effects model was used to determine the heterogeneity of the articles by calculating Cochran’s Q and *I*^2^. Acceptable heterogeneity of the results was defined as *I*^2^ ≤ 50%, whereas high heterogeneity was defined as *I*^2^ > 50%. When significant heterogeneity between studies was detected, subgroup analysis was conducted to identify possible sources of heterogeneity based on participant gender, sample size, geographic location, and study design. Publication bias was evaluated using Begg’s funnel plot and the Egger test. All statistical analyses were performed with Stata statistical software version 15.0.

## 3. Results

### 3.1. Eligible Studies

**Figure 1:**
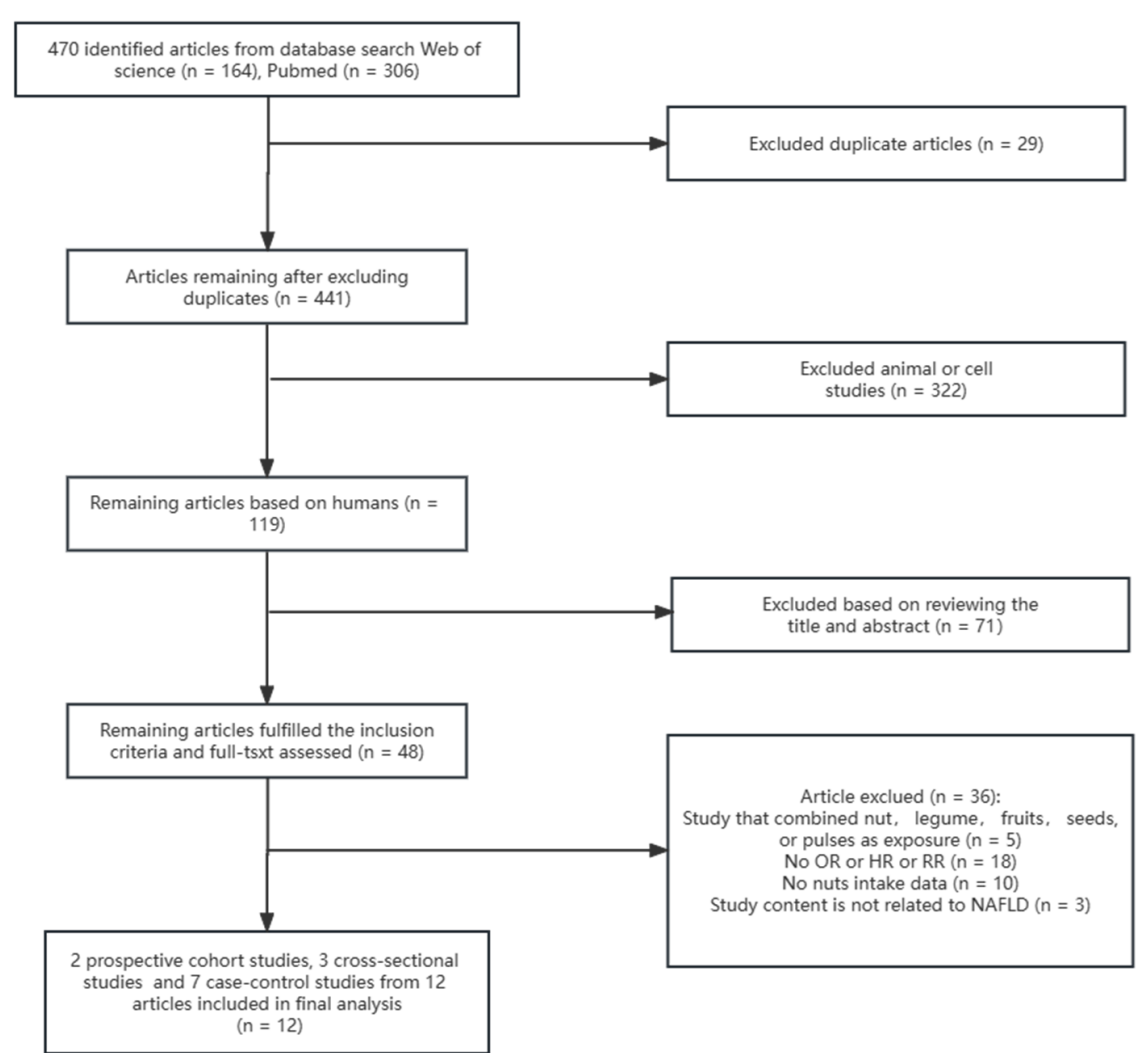
Flow diagram of the study selection. NAFLD, nonalcoholic fatty liver disease; HR, hazard ratios; RR, risk ratio; OR, odds ratio.

As shown in Figure 1, after an initial article search, we identified 470 studies and initially screened 441 studies after excluding 29 duplicate articles. After that, 322 articles on animals and cells were excluded. After applying the inclusion and exclusion criteria and reviewing the titles and abstracts, 71 articles were deemed ineligible for inclusion criteria. Finally, we reviewed the remaining 48 articles and found that 36 articles were unqualified. The reasons were as follows: (1) study that combined nut, legume, fruits, seeds, or pulses as exposure (n = 5), (2) no data on OR or HR or RR (n = 18), (3) no data on nut intake (n = 10), and (4) study content not related to NAFLD (n = 3). A total of 12 articles were ultimately included in the analysis, consisting of 2 prospective cohort studies, 3 cross-sectional studies, and 7 case–control studies.

### 3.2. Characteristics of Studies Included in the Meta-Analysis

The characteristics included in the study are shown in Table 1. The total number of participants in 12 articles included in the study is 92,621. We also calculated the number of participants in different study designs, prospective cohort study = 2, cross-sectional study = 3, case–control study = 7. Seven studies did not group men and women, while the remaining five studies calculated both the overall OR values and the OR values for men and women groups. According to the continental division, of the 12 studies, 8 were conducted in Asia, 3 were conducted in Europe, and 1 was conducted in North America. The number of participants in the study ranges from 155 people to over 30,000 people. The main adjusted confounding factors were age group, current job, body mass index (BMI), education level, exercise frequency, smoking status, and energy intake as collaborative variables. According to the score of NOS, the range of scores for 12 studies was 6–9 points, with an average score of 7.83, where the score for 7 articles was above the average score and was defined as a high-quality study.

### 3.3. Nut Intake and Total NAFLD Risk

**Figure 2:**
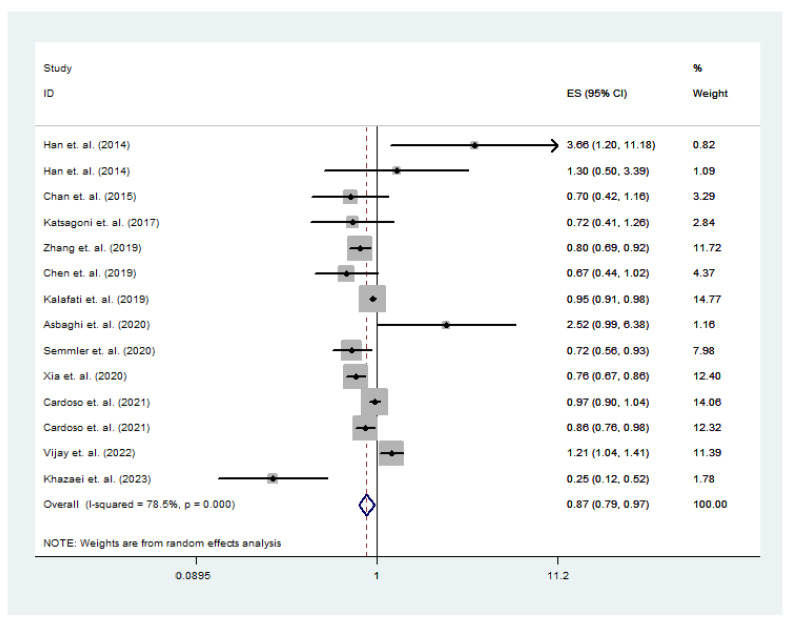
Association of nut intake with NAFLD according to a random effects meta-analysis. CI, confidence interval [2,19,20,21,22,23,24,25,33,34,35,36]. Black dots: odds ratio value.

The study used a random effects model to calculate the relationship between nut intake and total NAFLD risk. The meta-analysis results are shown in Figure 2. Due to two studies that did not report overall participant risk of consuming nuts and NAFLD, we included the male and female results reported in these studies in the overall data analysis [19,23]. The summary estimate of the study determined an OR of 0.87 (95% CI: 0.79–0.97, *p* < 0.001) between nut intake and total NAFLD risk, indicating a negative correlation. Heterogeneity was observed in the study (*p* < 0.001, *I*^2^ = 72.6%). Subgroup analysis explained the heterogeneity between studies by evaluating study design, gender, sample size, and nut intake. The asymmetric Egger linear regression test obtained a value of *p* = 0.463, and the Begg’s test was also used to obtain a value of *p* = 0.443. Therefore, there was no publication bias in this study.

### 3.4. Subgroup Analysis of the Effect of Total Nut Intake on the Risk of NAFLD

This study also conducted a subgroup analysis based on gender, region, and study design, and the results are shown in Table 2. The results of the subgroup analysis of articles from both sexes showed that the data on the impact of nut intake on the risk of NAFLD disease were not statistically significant (OR = 0.88; 95% CI: 0.72–1.07; *p* < 0.001). There was significant negative correlation between nut intake and NAFLD risk for women (OR = 0.83; 95% CI: 0.75–0.91) when compared with the risk for men (OR = 0.77; 95% CI: 0.56–1.0.8). In the subgroup analysis by region, we did not observe a correlation between nut intake and NAFLD risk in the Asian (OR = 0.88; 95% CI: 0.68–1.13; *p* < 0.001), Europe (OR = 0.84; 95% CI: 0.67–1.05; *p* = 0.07) and North American groups (OR = 0.92; 95% CI: 0.82–1.04; *p* = 0.107). In terms of study design, we observed a significant negative correlation between nut consumption and NAFLD in the cohort study (OR = 0.78; 95% CI: 0.69–0.88; *p* = 0.473) and cross-sectional studies (OR = 0.85; 95% CI: 0.74–0.98; *p* = 0.006). However, no significant correlation was observed in case–control studies (OR = 1.05; 95% CI: 0.83–13.32; *p* < 0.001). Finally, we also conducted a subgroup analysis based on the number of participants, with 1000 people as the grouping markers. Compared to the group with <1000 participants (OR = 0.93; 95% CI: 0.61–1.43; *p* < 0.001), the group with ≥1000 participants (OR = 0.87; 95% CI: 0.76–0.99; *p* < 0.001) had a significantly stronger correlation between the protective effect of consuming nuts on NAFLD.

## 4. Discussion

Nuts are rich in fiber, monounsaturated fatty acids (MUFA), and polyunsaturated fatty acids (PUFA), which could effectively prevent cardiovascular diseases and chronic diseases such as diabetes [37,38]. Recently, there has been a growing interest in the health benefits of nut intake among the public [39,40,41]. Currently, some studies have pointed out that nut intake had a significant effect on preventing NAFLD. In our meta-analysis, which included data from more than 90,000 people and had statistical capabilities, we found a negative correlation between nut consumption and the risk of NAFLD.

We have attempted to propose some mechanisms to explain the relationship between nut intake and NAFLD. The first mechanism involved antioxidant activity and inhibition of oxidative stress reactions. Nuts are rich in antioxidants and unsaturated fats [42], such as MUFA and PUFA [43,44], which were believed to help prevent NAFLD [45,46]. Van Name et al. indicated that a low *n*-6: *n*-3 PUFA diet could upturn the metabolic phenotype of adolescents with fatty liver [47]. A case–control study showed that *n*-6 PUFA can reduce liver fat and moderately improve metabolic status without losing weight [48]. Polyphenols, tocopherols, phytosterols (β-sitosterol), and other compounds in nuts have been proven to have antioxidant and protective effects against oxidative stress [49,50,51]. A study by Abenavoli et al. reported that different types of polyphenols (bergamot polyphenol fraction, resveratrol, and green tea polyphenols) can effectively reduce the pathological characteristics of NAFLD [52]. The inhibitory effect of nuts on oxidative stress can effectively reduce the risk of NAFLD [53].

In addition, consuming nuts can affect the risk of NAFLD through its effect on weight control. NAFLD is the most common liver manifestation of metabolic syndrome, closely related to obesity and insulin resistance [54,55]. Weight control and weight loss could alleviate NAFLD [56]. Nut intake has numerous benefits for weight control and weight loss [57]. A recent randomized controlled study found that consuming pistachios could result in a reduction in BMI (−4.9 [0.6] %) and waist circumference (*p* < 0.05) [58]. Nuts are rich in unsaturated fatty acids, dietary fiber, plant protein, and other prebiotic substances that are inaccessible nutrients [7]. Tindall et al. found that tree nuts may affect obesity by controlling appetite, replacing unfavorable nutrients, increasing availability of metabolizable energy, having the anti-obesity effects of bioactive compounds, and improving gut microbiome function [59]. Multiple studies have shown that nut consumption was negatively correlated with metabolic syndrome (MetS) and obesity [60,61,62]. Therefore, the intake of nuts could effectively control weight, reduce obesity risk, and, subsequently, reduce the likelihood of developing NAFLD [53].

Finally, the anti-inflammatory effect of nuts is also an important mechanism for achieving protective effects on a fatty liver. NAFLD is a general term that encompasses nonalcoholic fatty liver (NAFL) to nonalcoholic steatohepatitis (NASH) [63]. Inflammation is considered a key driver of NAFLD [64]. Liver enzymes are important biomarkers of liver diseases and can reflect NAFLD and liver injury [65]. A number of chemicals in nuts have anti-inflammatory effects, such as tocotrienols and ellagic alpha-linoleic acid (ALA) [66,67,68]. Leung et al. pointed out that ALA in nuts can reduce the production of pro-inflammatory mediators in the liver, demonstrating the regulation of anti-inflammatory lipid mediators [69]. Gu et al. indicated that ellagic acid could reduce liver malondialdehyde content and tumor necrosis factor alpha (TNF-α) and that the levels of alanine aminotransferase and aspartate transferase in the serum have anti-inflammatory effects [70]. Nut intake could reduce the concentration of liver enzymes and reduce the risk of NAFLD by inhibiting the occurrence of hepatitis [20].

Our final study showed that consuming nuts can effectively reduce the risk of NAFLD (OR = 0.87; 95% CI: 0.79–0.97; *p* < 0.001). However, a study by Han et al. reported that nuts do not have a protective effect towards NAFLD in both males and females (male: OR = 3.66; 95% CI: 1.20–11.18; female: OR = 1.30; 95% CI: 0.50–3.39) [23]. The research results of Asbaghi et al. (OR = 2.52; 95% CI: 0.99–6.38) and Vijay et al. (OR = 1.21; 95% CI: 1.04–1.41) also showed that nuts cannot reduce the risk of NAFLD [24,25]. Nuts are generally high in oil content and calories [24]. The excessive accumulation of fat in liver cells is the main characteristic of NAFLD, and lipid accumulation can exacerbate liver aging [71,72]. This may be one of the reasons why nut intake could increase the risk of NAFLD. It should be noted that some nuts, such as pistachios, may be contaminated with aflatoxin during processing or while being kept in improper storage environments [73]. Chronic aflatoxin poisoning may also lead to liver damage, leading to an increased risk of NAFLD [74].

Our meta-analysis has several strengths. First, it includes a large sample size of 92,621 participants and provides a comprehensive analysis of the effect of edible nuts on NAFLD. Previous meta-analyses on nuts have primarily focused on their relationship with cancer incidence and mortality rates. Second, we conducted a stratified analysis to investigate the impact of various confounding factors on the relationship between nut consumption and NAFLD risk. In addition, we attempted to propose two potential mechanisms to explain the protective effect of nuts on NAFLD. To the best of our knowledge, our study is the first meta-analysis to investigate the relationship between nut consumption and the risk of NAFLD, which is a significant contribution to the field.

However, when interpreting our findings, we must also consider its potential limitations. First, the number of studies analyzing the relationship between nuts and NAFLD was limited, with only 12 studies meeting our inclusion criteria. Due to the differences in study design, population characteristics, and other factors, there was a high degree of heterogeneity among the studies. In addition, the limited number of studies included in subgroup analyses may have led to biased results. Finally, as participants’ daily intake of food was not limited to nuts, but also contained other vegetables, fruits, and so on, it is possible that the potential impact of other dietary factors on the risk of NAFLD was not fully controlled for in the included studies, despite attempts to adjust for confounding variables. However, the protective effect of specific nuts on the liver could not be investigated because there were few studies. This will also be the focus of our future studies.

## 5. Conclusions

Our findings suggested a significant association between nut intake and a reduced risk of NAFLD, indicating a protective effect. Although gender-specific data were limited, our findings suggested that nuts may have a stronger protective effect towards NAFLD in women. Despite the growing evidence of the health benefits of nuts, research on the relationship between nuts and NAFLD remains limited. To our knowledge, our study is the first meta-analysis to investigate the impact of nut consumption on NAFLD risk, and we proposed two potential mechanisms to explain the protective effect. Nuts could be an important dietary component in the prevention of NAFLD, and further research is needed to better understand the protective effects of nuts on NAFLD and the relationship between other food types and NAFLD.

## Figures and Tables

**Table 1:** General characteristics of the included studies.

Studies	Study Design	Location	Years Enrolled	Age Range (Years)	Gender	Sample Size	Adjustment Variables	NOS
Han et al. (2014) [23]	Case–control Study	Korea	2014	20–69	Male/female	348	Age group, current job, education level, exercise frequency group, smoking, and energy intake.	6
Chan et al. (2015) [33]	Cross-sectional Study	China	2008–2010	19–72	Male/female	797	BMI, smoker status, drinker status, central obesity, triglyceride > 1.7 mmol/L, reduced HDL cholesterol, hypertension, impaired fasting glucose or diabetes, and the *PNPLA3* genotypes.	8
Katsagoni et al. (2017) [34]	Case–control Study	Greece	2013–2015	18–67	Male/female	155	Age, sex, waist circumference, HOMA-IR, adiponectin, and TNF-a.	7
Zhang et al. (2019) [21]	Prospective Cohort Study	China	2013–2016	≥18	Male/female	33,150	Age, gender, BMI, smoking status, alcohol drinking, education level, occupation, household income, physical activity, family history of disease, history of hypertension, total energy intake, eicosatetraenoic acid + docosahexaenoic acid intake, soft drink intake, three main dietary pattern scores, and potential intermediates of the nut–NAFLD association.	9
Kalafati et al. (2019) [22]	Case–control Study	Greece	2012–2015	≤65	Male/female	351	Age, sex, BMI or energy intake, smoking, and PAL.	7
Chen et al. (2019) [35]	Case–control Study	China	2015–2017	18–70	Male/female	1068	Age, income, smoking, educational level, tea drinking, occupation, marital status, BMI, physical activity, diabetes, hypertension and hyperlipidemia, and MUFA and PUFA intake.	8
Asbaghi et al. (2020) [24]	Case–control Study	Iran	2015	18–75	Male/female	999	Age, gender, BMI, alcohol drinking, smoking, diabetes, physical activity, and energy intake.	7
Semmler et al. (2020) [20]	Prospective Cohort Study	Austria	2010–2019	58.5 ± 9.8	Male/female	4655	Age, sex, BMI, metabolic syndrome, hepatic steatosis, alcohol drinking, intake of fast food, vegetables, fruits, sweets, red and processed meat, white meat, fish, coffee, and consumption of SSB.	9
Xia et al. (2020) [2]	Prospective Cohort Study	China	2013–2016	Na	Male/female	23,529	Age, sex, BMI, diabetes, hypertension, hyperlipidemia, physical activity, educational level, income, smoking, drinking, employment status, energy intake, total carbohydrate intake, total fat intake, sweet food intake, red meat intake, white meat intake, DHA + EPA intake, family history of CVD, hypertension, and diabetes.	9
Cardoso et al. (2021) [19]	Cross-sectional Study	USA	2005–2018	≥18	Male/female	25,360	Age, sex, smoking, HEI-2015, physical activity, history of CVD, and HbA1c.	9
Vijay et al. (2022) [25]	Case–control Study	India	2013–2016	≥25	Male/female	1966	Age, gender, and weight.	7
Khazaei et al. (2023) [36]	Case–control Study	Iran	2018–2019	average 42.7	Male/female	243	Age, sex, energy intake, physical activity, marital status, education, supplement use, drug use, smoking status, fat intake, carbohydrate intake (continuous), and BMI.	8

NOS, Newcastle-Ottawa Scale; BMI, body mass index; PAL, physical activity level; SSB, sugar-sweetened beverages; MUFA, monounsaturated fatty acids; PUFA, polyunsaturated fatty acids; DHA, docosahexaenoic acid; EPA, eicosapentaenoic acid; HEI-2015, Healthy Eating Index 2015; CVD, cardiovascular disease; HbA1c, Hemoglobin A1c. [2,19,20,21,22,23,24,25,33,34,35,36].

**Table 2:** Subgroup analysis of nut and NAFLD risk.

	Nuts		
	No. of Study	OR (95% CI)	*I* ^2^
**Sex**			
Male	5	0.77 (0.56, 1.08)	84.5%
Female	5	0.83 (0.75, 0.91)	2.8%
All	7	0.88 (0.72, 1.07)	82.6%
**Region**			
Asia	9	0.88 (0.68, 1.13)	83.7%
Europe	3	0.84 (0.67, 1.05)	62.4%
North America	2	0.92 (0.82, 1.04)	61.6%
**Study design**			
Prospective cohort study	2	0.78 (0.69, 0.88)	0.0%
Cross-sectional study	4	0.85 (0.74, 0.98)	76.2%
Case–control study	8	0.94 (0.73, 1.23)	80.6%
**Sample size**			
<1000	7	0.93 (0.61, 1.43)	76.2%
≥1000	7	0.87 (0.76, 0.99)	82.1%

CI, confidence interval; OR, odds ratio.

## Data Availability

The data presented in this study are available on request from the corresponding author.
